# The effects of *Viburnum opulus* L. fruit powder on the performance, egg traits, and egg cholesterol of laying Japanese quails

**DOI:** 10.5194/aab-69-507-2026

**Published:** 2026-09-03

**Authors:** Ayşe Gül Filik, Gökhan Filik, Olgay Kaan Tekin

**Affiliations:** 1 Kırşehir Ahi Evran University, Agriculture Faculty, Agricultural Biotechnology Department, TR40100, Kırşehir, Türkiye; 2 Filik Nanobioagrotech R&D and Technology Inc., TR41470, Kocaeli, Gebze, Türkiye

## Abstract

This study was conducted to evaluate the potential use of *Viburnum opulus* L. (VO) fruit as a phytogenic feed additive in the diets of Japanese quails (*Coturnix coturnix japonica*) and its effects on egg quality parameters and egg yolk cholesterol concentration. A total of 176 Japanese quails aged 30 weeks were used in the study. They were allocated into 4 main groups with 11 replicates, each consisting of 3 females and 1 male. The experimental groups consisted of the control (VOFP0) and the VOFP5, VOFP10, and VOFP15 groups, which received the basal diet supplemented with *Viburnum opulus* L. fruit powder (VOFP) at 0, 5, 10, and 15 g kg^−1^, respectively. The experiment lasted for 8 weeks. According to the results, dietary VOFP supplementation had no statistically significant effects on the quails' body weight, body weight gain, and feed conversion ratio (FCR) ( P>0.05 ). The absence of adverse effects on growth performance parameters suggests that VOFP was well tolerated under the conditions of this study. The lowest total feed consumption was recorded in the VOFP15 group, whereas the addition of VOFP5 resulted in the highest total egg weight (1828.60 g). Evaluation of egg quality parameters showed that VOFP supplementation improved selected internal egg quality parameters.

The dose of VOFP5 significantly increased egg yolk height and yolk index compared with the control group ( P<0.01 ). Regarding shell characteristics, VOFP supplementation reduced shell weight, whereas no adverse changes were observed in shell thickness and breaking resistance. Egg yolk cholesterol concentration showed VOFP supplementation numerical reductions at several sampling points; however, a statistically significant decrease was observed only in the sixth week in the VOFP15 group compared with the control group ( P<0.05 ).

In conclusion, the inclusion of VOFP5 in quail diets improved selected egg internal quality parameters, while VOFP15 showed a potential cholesterol-modulating effect, with a significant reduction observed only in the sixth week of the experiment. Considering the limited information available on VOFP supplementation in poultry nutrition, these findings provide preliminary evidence regarding the potential use of VOFP as a phytogenic feed additive and support further investigations into its effects on egg quality characteristics and potential functional properties of eggs.

## 1 Introduction

The modern livestock sector is striving to meet the increasing demand for animal protein associated with global population growth while facing major challenges, including rising feed costs and the sustainability of feed resources (Gok, 2023). In addition to economic pressures, the use of synthetic feed additives and chemical growth promoters in animal production has received increasing attention due to concerns regarding production costs and consumer preferences for more natural production systems (Wójcik-Bojek et al., 2021; Ozturkoglu-Budak et al., 2026). Today, consumers increasingly prefer animal products derived from animals fed with natural and reliable feed sources in accordance with the “clean label” concept (Erdal et al., 2022; Ozturkoglu-Budak et al., 2026). This approach is based on the concept that improved animal nutrition may contribute to better animal performance and product quality characteristics, potentially enhancing the functional value of animal-derived foods (Zakłos-Szyda et al., 2019; Mazur et al., 2021; Erdal et al., 2022).

In animal production, the incorporation of natural antimicrobial agents and plant-based antioxidants into diets is of strategic importance for managing diet costs and improving product quality (Wójcik-Bojek et al., 2021; Erdal et al., 2022). Plant-derived phytochemicals have been reported to possess antioxidant and biological activities that may contribute to maintaining animal health and improving product quality (Eken et al., 2017; Erdal et al., 2022; Juhnevica-Radenkova et al., 2024). In this context, the investigation of alternative plant sources with a rich bioactive component profile has become a critical requirement for both animal health and the products free from drug residues (Paşayeva et al., 2021; Sahin et al., 2022; Juhnevica-Radenkova et al., 2024).

At the center of this search is *Viburnum opulus* L. (VO), one of Türkiye's native plant resources with considerable phytochemical potential (Koparal, 2019; Celik, 2025). Belonging to the Adoxaceae family, this plant grows naturally in Europe, North Africa, and northern Asia, as well as in Türkiye, particularly in the central Anatolia region (Kayseri and its surroundings, Sivas, Tokat, Kırşehir, Ankara) (Baytop, 1999; Arslan et al., 2018; Polat et al., 2021; Celik, 2025). VO fruits are characterized by high concentrations of polyphenols, flavonoids, tannins, carotenoids, and vitamin C (Boyacı et al., 2016; Kajszczak et al., 2020; Zakłos-Szyda et al., 2020). Chlorogenic acid, which constitutes a dominant portion of the fruit's total phenolic profile (54 % to 96 %), is considered one of the principal compounds contributing to the plant's biological activity as a powerful antioxidant and antimicrobial agent (Kraujalytė et al., 2013; Barak et al., 2019; Zakłos-Szyda et al., 2019; Velioglu et al., 2006). Furthermore, modern studies have reported the positive effects of VO on regulating lipid metabolism, reducing free fatty acids, and cholesterol homeostasis (Zakłos-Szyda et al., 2019; Paşayeva et al., 2021).

Japanese quails (*Coturnix coturnix japonica*) are widely used in poultry nutrition studies because of their short generation interval, high egg production capacity, and suitability for evaluating dietary interventions. Although the biological activities of *Viburnum opulus* L. have been extensively investigated in relation to human health and pharmacological applications (Kajszczak et al., 2020; Sharifi-Rad et al., 2021), information regarding its potential use as a phytogenic feed additive in poultry nutrition remains limited. Therefore, the present study aimed to evaluate the effects of dietary *Viburnum opulus* L. fruit powder (VOFP) supplementation at different inclusion levels on growth performance, feed consumption, egg production performance, egg quality characteristics, and egg yolk cholesterol concentration in laying Japanese quails. The findings provide preliminary insights into the potential application of VOFP as a plant-based dietary additive in poultry production.

## 2 Materials and methods

The primary experimental material, VOFP, was harvested from the research and application field of Kırşehir Ahi Evran University after reaching full maturity. The fruits were cleaned, air-dried for 1 week, and then thoroughly dried in a ventilated oven at 65 °C for 48 h. A total of 176 Japanese quails (*Coturnix coturnix japonica*) of similar body weight, aged 30 weeks, were used as animal material in the study (132 females, 44 males). The cage was considered the experimental unit for all performance- and production-related measurements because dietary treatments were applied at the cage level. Each replicate consisted of 1 cage containing 3 female and 1 male quail, resulting in 11 independent replicates per dietary treatment. When forming the experimental groups, the birds were randomly allocated into 11 replicates of 4 main groups, with 3 females and 1 male in each cage. Therefore, all performance and egg production parameters were evaluated based on the mean values obtained from each replicate cage. The study lasted a total of 8 weeks (56 d). All experimental procedures were approved by the Local Ethics Committee for Animal Experiments at Kırşehir Ahi Evran University (decision no. 19-1, dated 2 October 2017).

The experimental treatments consisted of the control group (basal diet, VOFP0) as well as VOFP5 (basal diet + 5 g kg^−1^ VOFP), VOFP10 (basal diet + 10 g kg^−1^ VOFP), and VOFP15 (basal diet + 15 g kg^−1^ VOFP). The inclusion levels of VOFP (5, 10, and 15 g kg^−1^ diet) were selected to represent a safe and practical supplementation range for evaluating its potential effects in laying Japanese quails. As with other phytogenic feed additives, graded inclusion levels were preferred rather than applying high doses directly. The selected levels were determined considering previous experiences with plant-based feed additives and the need to evaluate possible dose-dependent responses without exceeding practical applicable supplementation range. The standard basal diet used was formulated to meet the nutritional requirements of quails, containing 177 g kg^−1^crude protein and 2800 kcal kg^−1^metabolizable energy (ME) (Table 1). The dried samples were ground into powder form using an ultra-centrifuge mill (Retsch ZM 200) with a 1 mm sieve to ensure homogeneous mixing into the diet. The dry matter, crude protein, crude fat, crude fiber, and ash contents of the diet were determined according to AOAC (2006) standard procedures.

**Table 1:** Raw material composition and nutrient content of the standard cage layer feed to be used in the experiment.

Feed ingredients	g kg^−1^	Calculated nutrient content	(%)
Corn	518.03	ME (kcal kg^−1^)	2800
Soybean meal (44 %)	212.93	Crude protein	17.70
Sunflower meal	93.55	Crude ash	12.7
Meat and bone meal	60.00	Crude fiber	4.57
Vegetable oil	35.88	Lysine	0.87
NaCl	2.72	Methionine + cysteine	0.66
DCP	2.46	Calcium	3.60
Marble dust	70.57	Available phosphorus	0.49
Methionine (HA)	0.36		
Vitamin–mineral premix^1−2^	3.50		
Total	1000.00		

^1^ Provided per kilogram of diet: retinyl acetate, 9012 IU; cholecalciferol, 1500 IU; DL- α -tocopheryl acetate, 7.5 IU; thiamin, 0.6 mg; riboflavin, 4.8 mg; pyridoxine hydrochloride, 1.5 mg; cyanocobalamin, 0.009 mg; calcium-D-pantothenate, 7.5 mg; folic acid, 0.15 mg; niacin, 20 mg.^2^ Provided per kilogram of diet: copper (CuSO ${}_{4}⚫$ 5H_2_O), 6 mg; iron (FeSO ${}_{4}⚫$ H_2_O), 60 mg; zinc (ZnSO ${}_{4}⚫$ H_2_O), 80 mg; manganese (MnSO ${}_{4}⚫$ H_2_O), 60 mg; selenium (NaSeO_3_), 0.3 mg; iodine (KI), 0.35 mg.

During the experiment, a 16 : 8 light–dark photoperiod was applied; feed and water were provided ad libitum to the quails. Body weight (BW), feed intake (FI), and feed conversion ratio (FCR) were monitored weekly. Eggs were collected twice daily (at 09:00 and 15:00; all instances are in local time), and productivity was recorded on a “quail-day” basis.

Eggs collected on the third day of each week were subjected to laboratory analysis to determine egg quality parameters. Among the external quality parameters, egg weight was measured on a scale with 0.1 g precision, while width and length were measured using a digital caliper (Filik et al., 2020). The shape index ((egg width / egg length) × 100) was calculated using the formula. Shell thickness (mm) was measured using a micrometer at the pointed, middle, and blunt ends of the egg, and the average was taken. Egg breakage resistance (cm^2^g^−1^) was determined using a Brookfield CT3 texture analyzer (Filik, 2009). For internal quality parameters, eggs were broken, and the heights of the white and yolk were measured with a micrometer; white and yolk indices were calculated. The Haugh unit, accepted as the internal quality standard, was determined using the formula (100 log (solid white height + 7.57 - 1.7 × egg weight^0.37^)), as also stated by Filik (2009). The yolk color ( L *, a *, b *, chroma, and $h^{\circ }$ values) was measured spectrophotometrically using a Konica Minolta CR-410 Chroma Meter (Filik et al., 2018).

To monitor the effects of the VOFP supplement on cholesterol metabolism, cholesterol analysis was performed on eggs collected on the first day and during the second, fourth, sixth, and eighth weeks as well as the final day of the experiment. After boiling the eggs for 10 min, the yolks were separated and homogenized, followed by extraction with isopropyl alcohol. The resulting supernatants were measured at 520 nm wavelength using a spectrophotometer to calculate cholesterol concentrations. This method is based on the procedures of Chowdhury and Smith (2002), who investigated the effects of phytogenic additives.

The data were analyzed using the general linear model (GLM) procedure of SAS (1997), considering the cage as the experimental unit. Dietary treatment was included as the main effect in the statistical model. The replicate cage means were used for performance, egg production, and egg quality measurements to avoid pseudo-replication resulting from multiple animals within the same cage. Orthogonal polynomial contrasts (linear, quadratic, and cubic effects) were applied to determine dose–response relationships among VOFP supplementation levels (Düzgüneş et al., 1987). Egg yolk cholesterol concentrations were measured at different sampling times during the experimental period, and the results were evaluated according to the dietary treatment effects observed at each sampling period. The statistical significance level was set at P<0.05 and P<0.01 . The difference between the groups was made using Duncan's multiple comparison method (Genç and Soysal, 2018).

## 3 Results

At the end of the experiment, dietary VOFP supplementation was not found to have a statistically significant effect on body weight (BW), feed conversion ratio (FCR), and body weight gain (BWG) ( P>0.05 ). However, among the treatment groups in terms of total feed intake (TFI), the highest feed intake was observed in the VOFP5 group at 7888.0 g, while the lowest intake was recorded in the VOFP15 group at 7115.6 g ( P<0.05 ) (Table 2).

**Table 2:** The effects of different levels of VOFP supplementation on body weight gain, feed intake, and feed conversion ratio of Japanese quail layers (g kg^−1^).

Parameters	Groups (g kg^−1^)				SED	P	Effects^*^		
	VOFP0	VOFP 5	VOFP 10	VOFP 15			L	Q	C
Initial BW (g)	166.96	165.65	164.88	164.58	1.47	0.94	0.5460	0.8630	0.9960
Final BW (g)	172.96	171.65	170.88	170.58	0.98	0.83	0.3690	0.7960	0.9940
BWG (g)	6.00	6.00	6.00	6.00	0.51	1.00	1.0000	1.0000	1.0000
Feed intake ( $g\;d^{-7}$ )									
Week 1	606.40	606.00	585.20	581.60	49.27	1.00	0.8410	0.9840	0.9060
Week 2	737.30	814.50	891.40	736.00	72.02	0.85	0.9080	0.4210	0.7230
Week 3	650.10	822.80	641.60	920.00	79.43	0.55	0.3910	0.7400	0.2500
Week 4	787.20	572.70	652.90	647.70	88.64	0.86	0.6830	0.5570	0.6190
Week 5	792.20	498.90	737.00	715.50	84.88	0.55	0.9920	0.4340	0.2890
Week 6	689.00	552.10	757.30	866.80	85.61	0.61	0.3660	0.4810	0.5580
Week 7	576.60	591.40	654.00	551.30	86.33	0.98	0.9860	0.7330	0.7840
Week 8	689.30	750.40	602.10	646.30	87.60	0.93	0.7290	0.9610	0.5970
TFI ( $g\;d^{-56}$ )	7478.6ab	7888.0a	7287.4ab	7115.6b	120.01	0.15	0.1260	0.2320	0.1880
AFI (g per 4 birds $d^{-7}$ )	155.80ab	164.33a	151.82ab	148.24b	2.50	0.15	0.1260	0.2320	0.1880
Feed conversion ratio (egg yield/feed intake, 7 d)									
Week 1	8.11	7.84	7.43	8.36	0.87	0.98	0.9630	0.7270	0.8520
Week 2	3.69	3.66	4.04	5.56	0.45	0.42	0.1460	0.3900	0.8530
Week 3	3.66	3.84	3.38	6.34	0.57	0.31	0.1580	0.2340	0.4220
Week 4	3.88	2.65	3.92	5.07	0.50	0.36	0.3040	0.2430	0.5420
Week 5	4.16	2.79	4.55	4.89	0.53	0.46	0.4280	0.4290	0.3310
Week 6	4.30	3.10	5.47	5.58	0.52	0.26	0.2120	0.5390	0.2100
Week 7	3.08	3.15	3.75	5.02	0.58	0.62	0.2200	0.6010	0.9770
Week 8	3.78	3.70	4.43	5.88	0.58	0.54	0.1910	0.5080	0.9880
AFCR	4.86	4.83	5.14	5.84	0.32	0.65	0.2620	0.5660	0.9810

a–b: means in the same row bearing different superscripts differ significantly. BW: body weight; BWG: body weight gain; TFI: total feed intake; AFI: average of feed intake; AFCR: average feed conversion ratio. SED: standard error of the difference between the means.^*^ Effects. L: linear; Q: quadratic; C: cubic.

Dietary VOFP supplementation had no statistically significant effect on average egg weight, total egg weight, and total egg number during the 8-week experimental period ( P>0.05 ) (Table 3). However, the highest total egg weight was observed in the VOFP5 group (1828.60 $g\;d^{-56}$ per three quails). The VOFP5 group (53.00 eggs) showed higher egg production in the afternoon (15:00) compared to the control (39.20 eggs) and VOFP15 (30.50 eggs) groups.

**Table 3:** The effects of different levels of VOFP on egg weight and yields of Japanese quail layers (g kg^−1^).

Parameters		Groups (g kg^−1^)				SED	P	Effects^*^		
		VOFP0	VOFP 5	VOFP 10	VOFP 15			L	Q	C
Average of egg weight	Week 1	12.48	13.55	13.33	12.8	0.24	0.38	0.720	0.100	0.653
	Week 2	12.68	13.81	13.58	13.03	0.19	0.16	0.630	0.032	0.551
	Week 3	13.01	13.52	13.61	13.29	0.18	0.66	0.581	0.261	0.998
	Week 4	13.34	13.02	13.75	13.11	0.14	0.26	0.973	0.561	0.059
	Week 5	13.55	13.19	13.07	13.17	0.17	0.77	0.408	0.511	0.994
	Week 6	13.37	13.48	13.52	12.87	0.19	0.58	0.381	0.309	0.717
	Week 7	13.76a	13.56a	13.43ab	12.60b	0.15	0.05	0.011	0.313	0.574
	Week 8	13.70	12.43	13.69	13.11	0.32	0.48	0.860	0.602	0.142
Total egg weight	Week 1	197.00	216.10	188.64	192.09	11.36	0.84	0.678	0.733	0.455
	Week 2	218.05	250.40	234.18	191.36	10.21	0.23	0.294	0.074	0.813
	Week 3	210.00	233.90	219.55	177.55	10.13	0.26	0.221	0.112	0.908
	Week 4	210.91ab	236.80a	214.82a	165.27b	8.02	0.03	0.031	0.024	0.781
	Week 5	193.18	230.40	202.82	178.27	11.08	0.43	0.466	0.172	0.502
	Week 6	183.73	225.00	191.55	165.82	11.08	0.32	0.381	0.139	0.416
	Week 7	189.00ab	228.80a	171.18ab	156.73b	10.31	0.10	0.099	0.196	0.140
	Week 8	171.00	207.20	173.45	152.30	11.11	0.41	0.378	0.210	0.417
	Morning (09:00)	4862.00	4786.00	4609.00	4612.00	509.42	1.00	0.839	0.969	0.950
	Afternoon (15:00)	1098.3ab	1497.7a	1441.2a	932.4b	81.90	0.06	0.438	0.010	0.996
Total egg weight (56 d per three quails)		1572.90	1828.60	1596.20	1440.80	70.37	0.32	0.330	0.158	0.381
Average weight (56 d per three quails)		28.09	32.65	28.50	25.73	1.26	0.32	0.330	0.158	0.381
Egg number	Morning (09:00)	91.67	98.40	100.27	97.70	4.72	0.94	0.658	0.638	0.992
	Afternoon (15:00)	39.20	53.00	–	30.50	3.43	0.35	–	–	–
Total		119.91	136.63	118.27	110.80	5.09	0.37	0.329	0.249	0.326

a–b: means in the same row bearing different superscripts differ significantly. SED: standard error of the difference between the means.^*^ Effects. L: linear; Q: quadratic; C: cubic

The egg quality results are presented in Table 4. Differences among the treatment groups in terms of shell weight, percentage (%) of shell weight, and egg yolk height were found to be statistically significant ( P<0.01 ).

**Table 4:** The effects of different levels of VOFP on the egg quality parameters of Japanese quail layers (g kg^−1^).

Parameters	Groups (g kg^−1^)				SED	P	Effects^*^		
	VOFP0	VOFP 5	VOFP 10	VOFP 15			L	Q	C
Egg weight (g)	13.75	14.67	13.79	13.01	0.32	0.352	0.286	0.193	0.509
Shell weight (g)	1.39ab	1.22c	1.40a	1.31b	0.02	0.000	0.695	0.203	0.000
Shell weight (%)	10.09a	8.65b	10.16a	10.09a	0.14	0.001	0.223	0.018	0.001
Shape index (%)	79.13	79.11	79.03	79.41	0.32	0.976	0.791	0.751	0.852
Width (mm)	26.88	26.59	26.84	26.20	0.13	0.269	0.142	0.512	0.24
Length (mm)	33.97	33.62	33.97	33.04	0.19	0.268	0.155	0.452	0.242
Egg shell top (mm)	0.20	0.21	0.20	0.20	0.00	0.378	0.188	0.248	0.954
Egg shell mid (mm)	0.21	0.21	0.21	0.20	0.00	0.233	0.256	0.188	0.256
Egg shell bottom (mm)	0.21	0.21	0.21	0.20	0.04	0.34	0.464	0.106	0.675
Average thickness (mm)	0.21	0.21	0.21	0.20	0.00	0.251	0.24	0.129	0.516
Albumen weight (g)	8.08	9.35	8.13	7.74	0.32	0.298	0.425	0.193	0.243
Albumen weight (%)	58.75b	62.28a	58.94b	59.42ab	0.54	0.086	0.783	0.164	0.031
Albumen height (mm)	6.12	6.35	5.90	5.89	0.07	0.109	0.962	0.439	0.095
Albumen width (mm)	37.88	37.29	37.79	36.43	0.38	0.510	0.257	0.615	0.385
Albumen length (mm)	50.02	49.06	47.88	47.51	0.46	0.216	0.04	0.753	0.803
Albumen pH	8.68	8.58	8.64	8.55	0.04	0.641	0.361	0.939	0.366
Albumen index (%)	8.92	9.39	8.87	9.03	0.16	0.647	0.892	0.629	0.241
Yolk weight (g)	4.28a	4.10ab	4.26a	3.97b	0.05	0.091	0.078	0.571	0.076
Yolk weight (%)	31.16	29.08	30.90	30.49	0.45	0.359	0.961	0.350	0.128
Yolk height (mm)	13.14ab	13.55a	13.07ab	12.69b	0.08	0.006	0.015	0.017	0.176
Yolk width (mm)	23.44	22.91	22.93	22.70	0.15	0.347	0.103	0.613	0.549
Yolk index (%)	56.14b	59.22a	59.10ab	55.94b	0.49	0.082	0.531	0.035	0.163
L *	71.60	72.76	72.24	71.59	0.35	0.592	0.866	0.205	0.629
a *	12.85	14.19	13.13	12.28	0.38	0.338	0.409	0.150	0.435
b *	18.25b	18.40ab	19.74a	18.62ab	0.24	0.124	0.250	0.190	0.092
Chroma	362.19	367.71	419.80	378.49	10.47	0.209	0.282	0.265	0.139
$h^{\circ }$	9.21	9.35	8.98	9.08	0.08	0.419	0.305	0.882	0.188
Haugh unit	96.37	97.00	95.24	95.73	0.40	0.433	0.305	0.927	0.198
Breaking resistance (cm^2^g^−1^)	1106.63	1228.84	1119.72	1063.01	35.69	0.404	0.452	0.212	0.374

a–b: means in the same row bearing different superscripts differ significantly. SED: standard error of the difference between the means.^*^ Effects. L: linear; Q: quadratic; C: cubic.

When shell characteristics were examined, VOFP supplementation reduced shell weight; the lowest shell weight (1.22 g) was observed in the VOFP5 group ( P<0.01 ). Shell thickness, however, was not affected by VOFP supplementation. Among the internal egg quality parameters, egg yolk height was highest in the VOFP5 group (13.55 mm), accompanied by an increase in yolk index ( P<0.01 ). Albumen weight (62.28 %) and albumen index (9.39 %) were also highest at the VOFP5 dose. Among the yolk color parameters, the b * value was highest (19.74) in the VOFP10 group. The Haugh unit and breaking resistance parameters were not statistically affected by the addition of VOFP ( P>0.05 ).

**Table 5:** Effect of VOFP on quail egg cholesterol concentration (g kg^−1^).

Parameters	Groups (g kg^−1^)				SED	P	Effects^*^		
	VOFP0	VOFP 5	VOFP 10	VOFP 15			L	Q	C
First day in the experiment	43.48	41.95	43.40	43.28	0.87	0.910	0.912	0.684	0.563
Second week	46.68	46.87	46.82	44.63	1.11	0.844	0.533	0.592	0.849
Fourth week	43.59	45.07	42.40	45.01	1.18	0.832	0.879	0.811	0.377
Sixth week	52.31a	44.25b	47.07ab	41.04b	1.27	0.028	0.011	0.689	0.081
Eighth week	46.79	45.51	47.52	43.64	0.80	0.373	0.300	0.417	0.204
Last day in the experiment	47.01ab	40.79b	49.62a	44.87ab	1.36	0.145	0.841	0.789	0.025
Average cholesterol	46.83	45.01	46.61	43.51	0.81	0.442	0.262	0.692	0.261

a–b: means in the same row bearing different superscripts differ significantly. SED: standard error of the difference between the means.^*^ Effects. L: linear; Q: quadratic; C: cubic.

The effects of VOFP supplementation on egg yolk cholesterol concentration are presented in Table 5. Although average egg yolk cholesterol concentration decreased numerically in the groups supplemented with VOFP throughout the experiment, this difference was not statistically significant ( P>0.05 ). However, a significant difference in egg yolk cholesterol concentration was observed in the sixth week of the experiment; the egg yolk cholesterol concentration, which was 52.31 mg g^−1^ in the control group, decreased to 41.04 mg g^−1^ in the VOFP15 group ( P<0.05 ). The significant reduction observed in the sixth week suggests a potential time-dependent effect of VOFP supplementation on egg yolk cholesterol concentration. However, this effect was not consistently observed throughout the entire experimental period.

## 4 Discussion

This study, which examined the effects of VOFP added to the diet on the performance and egg quality characteristics in quails, represents a relatively unexplored area in the literature on phytogenic feed additives. VO has traditionally been used in Anatolia, particularly in the Kayseri region, and has been associated with various traditional uses, including kidney health, digestive regulation, and antidiabetic applications (Gok, 2023; Celik, 2025). Recent studies indicate that the pharmacological effects of VO (such as anticancer, antidiabetic, and DNA interaction) have gained momentum in recent years (2024–2025) (Giritlioglu et al., 2025; Kaya et al., 2025). However, studies investigating the direct use of *Viburnum opulus* L. as a dietary additive in poultry nutrition are currently unavailable or very limited. Therefore, the present study provides initial data regarding the potential utilization of VOFP in Japanese quail nutrition. Based on performance parameters and metabolic tolerance data, VOFP supplementation did not cause a negative change in quail body weight and feed conversion ratio (FCR) (Table 2), suggesting that the tested inclusion levels were well tolerated by avian metabolism. Interestingly, the numerical decrease in feed intake at high doses such as 15 g kg^−1^ (Table 2) may be associated with the inhibitory effect of VOFP's acidic profile, resulting from its high organic acid content (especially malic and quinic acids), on palatability (Effatparvar et al., 2025). Free amino acids such as aspartic and glutamic acid, found in high concentrations in the fruit, may support protein metabolism, but the acidic nature of the fruit may limit consumption at high doses (Juhnevica-Radenkova et al., 2024).

When examining egg production and quality dynamics, the fact that VOFP did not cause a significant change in the total number of eggs (Table 4) indicates that the animals maintained their physiological peak production levels. However, the increase in egg yolk height and yolk index observed at a dose of VOFP5 in Table 4 ( P<0.01 ) is one of the principal findings of our study. Previous studies have reported that *Viburnum opulus* L. fruits contain various bioactive compounds, including phenolic compounds such as chlorogenic acid and catechin derivatives (Juhnevica-Radenkova et al., 2024; Effatparvar et al., 2025). These compounds have been associated with antioxidant activity and lipid-related biological effects in previous studies. However, the specific phytochemical composition of the VOFP used in the present study was not determined. Therefore, the possible contribution of these compounds to the observed changes in egg quality should be regarded as a hypothetical and requires further investigation rather than a confirmed mechanism. On the other hand, it has been reported that processing VO fruit using methods such as encapsulation increases the stability of bioactive compounds and preserves their functionality in food matrices (Ozturkoglu-Budak et al., 2026). Despite the decrease in shell weight (Table 4), the preservation of shell thickness indicates that VOFP did not adversely affect mineral metabolism.

The significant decrease in egg yolk cholesterol concentration observed in the VOFP15 group during the sixth week of the experiment suggests a potential time-dependent effect of VOFP supplementation on cholesterol metabolism (Table 5). Previous studies have reported that *Viburnum opulus* L. contains phenolic compounds associated with antioxidant activity and lipid metabolism. VO-derived phenolic compounds have been suggested to influence lipid accumulation and fatty acid metabolism through cellular mechanisms (Zakłos-Szyda et al., 2019). However, the specific phytochemical composition of the VOFP used in the present study and the related metabolic pathways were not evaluated. Therefore, the reduction observed in egg yolk cholesterol concentration should be interpreted as a potential effect of VOFP supplementation rather than a confirmed hypocholesterolemic mechanism. Further studies including phytochemical characterization, hepatic cholesterol metabolism markers, antioxidant status, and gut microbiota analyses are needed to clarify the mechanisms underlying these observations.

## 5 Conclusion

This study indicates that VOFP has potential as a phytogenic feed additive for modulating selected egg quality characteristics in quail nutrition. The absence of adverse effects on body weight, feed conversion ratio, and general performance parameters suggest that VOFP was well tolerated under the conditions of this study. The VOFP5 dose improved selected internal egg quality parameters, while bioactive compounds were reported in *Viburnum opulus* L.; however, the specific compounds responsible for these effects were not determined in the present study. Regarding cholesterol metabolism, although numerical reductions were observed in some sampling periods, a significant decrease was detected only during the sixth week in the VOFP15 group compared with the control group ( P<0.05 ). These findings suggest that VOFP may have potential as a phytogenic feed additive for improving selected egg quality characteristics; however, further studies are required to evaluate its possible contribution to functional egg production. Future studies including phytochemical characterization, antioxidant capacity evaluation, and physiological mechanism-based analyses will provide further insight into its biological effects.

## Data Availability

The original contributions presented in the study can be directed to the corresponding author.
